# A Peer Support Specialist–Delivered Sexual and Intimate Partner Violence Prevention Program for Women in Substance Use Treatment: Protocol for a Single-Arm Trial

**DOI:** 10.2196/68673

**Published:** 2025-08-08

**Authors:** Heidi M Zinzow, Irene Pericot-Valverde, Lauren Smalls, Madelyn G Brancato, Greyson Chapman, Allison Smith, Ava Thompson, Caroline Greco, Meghan Shank, Kacey Y Eichelberger, Kimbley Smith, Alain H Litwin

**Affiliations:** 1 Clemson University Clemson, SC United States; 2 Prisma Health Greenville, SC United States; 3 Phoenix Center Greenville, SC United States

**Keywords:** interpersonal violence, sexual violence, sexual assault, dating violence, partner violence, repeated exposure to violence, intervention, peer recovery coaches, addiction, substance use disorders, drug use

## Abstract

**Background:**

Women in substance use treatment are disproportionately affected by violence. Both a history of violence and substance use place women at risk for cumulative exposure to violence and adverse outcomes, including mental and physical health problems. Interventions are urgently needed to reduce these health disparities by preventing initial and repeated exposure to sexual and intimate partner violence among women with substance use disorders (SUDs). The Healthy Relationships and Interpersonal Violence Education (THRIVE) program adapts evidence-based strategies for this population and is informed by the information, motivation, behavioral skills theoretical model. Topics include the intersection of substance use and violence, consent, risk detection, protective behavioral strategies, and help seeking. THRIVE uses a novel approach by delivering the program via peer support specialists (PSSs), trained advocates in recovery from SUDs who can help overcome barriers to care, including stigma and accessibility.

**Objective:**

The first objective is to determine program acceptability and feasibility. The second objective is to determine the preliminary effectiveness of THRIVE, including its effect on violence-related knowledge and attitudes, protective behaviors, exposure to sexual and intimate partner violence, substance use, and mental health.

**Methods:**

The study entailed a single-arm trial of THRIVE with 71 women in behavioral and medication-assisted substance use treatment, recruited from 3 outpatient and residential treatment sites. Interview data assessing intervention acceptability and feasibility were collected from participants and PSSs. Participants completed assessments at 4 time points over 3 months (baseline, after the intervention, and 1- and 3-month follow-ups). Self-report questionnaires assessed (1) violence prevention knowledge, attitudes, and behaviors; (2) exposure to sexual and intimate partner violence; and (3) substance use and mental health. To determine acceptability and feasibility, both quantitative and qualitative data were collected on feasibility (recruitment and retention), adherence, and acceptability (engagement, perceived usefulness, barriers and facilitators to participation and adoption, and working alliance with PSSs).

**Results:**

Of the 92 women recruited and enrolled, 71 (77%) completed the intervention, 58 (63%) completed the 1-month follow-up, and 44 (48%) completed the 3-month follow-up between June 2024 and March 2025. The mean age of enrolled participants was 35 (SD 9.87) years, and the majority were White (n=79, 86%), followed by Black (n=4, 4%) and other racial and ethnic identities (n=7, 8%).

**Conclusions:**

THRIVE will address critical gaps in the field by (1) expanding violence prevention strategies to SUD treatment settings, (2) integrating sexual and intimate partner violence prevention, (3) incorporating a focus on illicit substance use, and (4) engaging PSSs to overcome barriers to care. The long-term objective of this project is to develop an accessible, scalable, and efficacious prevention program that reduces the incidence of exposure to sexual and intimate partner violence, substance use, and violence-related mental health disorders for women in substance use treatment.

**Trial Registration:**

ClinicalTrials.gov NCT06608979; https://clinicaltrials.gov/study/NCT06608979

**International Registered Report Identifier (IRRID):**

DERR1-10.2196/68673

## Introduction

### Interpersonal Violence History, Substance Use, and Health

A substantial proportion (60%-90%) of individuals in substance use treatment report a history of violence, with women being at greatest risk for initial and repeated exposure to interpersonal violence [1-3]. Interpersonal violence is an overarching term that encompasses both sexual violence (SV) and intimate partner violence (IPV) [4]. SV can be defined as nonconsensual sexual contact through physical force or inability to consent (eg, incapacitation due to drugs or alcohol). It can include harassment, unwanted contact, coerced sex, or attempted or completed rape and more recently has been expanded to include cyber-SV [5,6]. IPV often co-occurs with SV and includes physical violence, SV, stalking, and psychological aggression that occurs in the context of a current or former romantic relationship [7]. Given the high prevalence of interpersonal violence among women with substance use disorders (SUDs), there is an urgent need for interventions to prevent initial and repeated exposure to interpersonal violence in this population.

Interpersonal violence is a critical public health problem associated with a host of physical and mental health outcomes, including posttraumatic stress disorder (PTSD), depression, SUDs, chronic pain, gastrointestinal disorders, and a variety of chronic health conditions [8-10]. In particular, women in SUD treatment with a history of violence are more likely to experience relapse and poor recovery outcomes [11-13]. Both a history of violence and substance use place women at risk for exposure to cumulative interpersonal violence, and two-thirds of women with exposure to SV experience repeated exposure [1,14,15]. Substance use is frequently cited as a coping mechanism for managing posttraumatic stress and the aftereffects of exposure to violence and also places women at risk for exposure to interpersonal violence [1,14]; for example, research has indicated that individuals in treatment for co-occurring disorders such as PTSD and SUD are at greater risk for exposure to violence than individuals with SUD alone [16].

Despite the intersection of trauma and substance use problems, there is a disconnect between trauma-informed services and substance use treatment [17]. Substance use treatment can be defined as behavioral or pharmacological treatment provided by medical and mental health professionals for a range of SUDs (eg, alcohol, opioid, stimulant, polysubstance use) [18]. The Substance Abuse and Mental Health Services Administration (SAMHSA) has proposed a model of trauma-informed care to adopt in behavioral and substance use treatment facilities, further emphasizing the importance of an integrated approach to substance use treatment [19]. Although the acceptance of and evidence for integrated treatments for trauma and SUD have grown, studies indicate that less than half of SUD treatment facilities offer trauma-specific services [17,20,21]. The facilitators of adopting these models include state policies supportive of screening, insurance reimbursement, and evidence-based treatment for trauma [21]. Barriers to implementation include organizational factors such as the logistics of adopting integrated treatments within current service time frames and formats, mental health provider concerns about the timing of trauma treatment initiation or ongoing exposure to violence, lack of mental health provider training, and high case management needs in this population [22]. These studies have primarily focused on the provision of mental health services, and little is known about the promotion of violence prevention programs in SUD treatment settings. Without efficacious violence prevention programs integrated into these settings, women with SUDs will continue to experience trauma-related health disparities.

### Barriers to Care

In addition to a lack of access to integrated service delivery models, women with SUDs and a history of interpersonal violence face multiple barriers to engaging in trauma-informed services and mental health care. Barriers include stigma, lack of acknowledgment of violent incidents, mistrust in formal systems, being a member of a group considered oppressed or minoritized, problematic gender role norms, lack of available resources, and fears of negative consequences [23,24]. Studies indicate that individuals with a history of violence frequently experience multiple co-occurring mental health concerns and, in the face of other barriers to care, are unlikely to seek treatment [25]. Furthermore, individuals with a history of violence and substance use face specific barriers such as lack of acknowledgment of substance-involved sexual assaults as rape, perceived lower credibility by health care providers, and fears of facing criminal charges [23]. These barriers inhibit engagement with prevention and recovery, as well as curtail help-seeking behavior. Furthermore, the strain on health care systems to address the current mental health crisis has limited the availability of mental health professionals to deliver preventive care and clinical interventions [26,27].

### Potential of Peer Support Models to Advance Trauma-Informed Care

The SAMHSA model for trauma-informed care highlights the important role of peer support services in overcoming these barriers and in ensuring a continuum of care. Peer support specialists (PSSs) are individuals who have achieved stable recovery from mental health conditions [28]. Although the definitions of recovery differ between peer-certifying organizations, many align with SAMHSA’s definition, which describes recovery as a process of change to improve health and wellness and strive toward one’s full potential, rather than a symptom-free end state [29]. As such, recovery can be achieved via multiple pathways such as mental health treatment, family and peer support, and faith-based interventions [29]. PSSs use both their lived experience and relevant training to guide and mentor other individuals in treatment or recovery [30]. Peer support programs are based on the principles of mutual respect and support, empowerment, shared responsibility, and an empathy and connection that can be achieved outside of the constraints of a hierarchical (expert and patient) relationship [31]. These programs aim to reduce marginalization and stigma while offering an extension of mental health care beyond the bounds of formal services. PSSs not only offer a means of overcoming resource demands but can also address other significant barriers to care, such as stigma, mistrust in formal systems, and limited availability of mental health providers representing minoritized identities [32]. Peer norms are a significant predictor of substance use as well as both perpetration of and exposure to violence, and the involvement of peers as prevention educators could be especially fruitful in shifting these norms [33,34]. Violence prevention programs are sorely needed in substance use treatment settings, and PSSs can serve as an important resource for offering the tools to prevent initial and repeated exposure to interpersonal violence and the associated mental health sequelae among women with SUDs.

Peer support programs were integrated into formal mental health systems in the 1980s and currently represent one of the fastest growing sectors of the mental health workforce [30]. Research has established the value of PSS models for suicide prevention and the management of mental health conditions [35,36]. In addition, some violence prevention programs have used peers as advocates, with peers being defined as fellow students or adolescents [37,38]. However, studies have yet to evaluate PSS models for offering violence prevention services in various mental health care settings. Our program extends this promising model to implement needed prevention programming via trusted and trained peer facilitators in substance use treatment settings.

### Violence Prevention Programs

Numerous evidence-based programs for SV and IPV prevention have been developed and supported by prior research [39,40]. However, they have almost exclusively been delivered in college and school-based settings, and a recent review has indicated minimal changes in the overall prevalence of SV exposure and perpetration over time [41]. A recent meta-analysis showed that campus SV prevention programs have significant impact on rape myth acceptance, knowledge and attitudes toward sexual assault, knowledge of consent, and exposure to SV [39]. By contrast, a similar meta-analysis highlighted limited behavior change, despite changes in knowledge and attitudes [42]. Most of these programs are geared toward a broad audience of individuals at risk of experiencing SV, potential perpetrators, and bystanders. The enhanced assess, acknowledge, act model is one of the few models to show efficacy for specifically addressing risk for exposure to SV among women by using risk recognition, reducing barriers to active resistance, and engaging in protective strategies [43-45]. Participants demonstrated decreased rape myth acceptance, increased resistance strategies, and decreased incidence of completed rape at 6 months [43]. To our knowledge, very few programs address alcohol or other substance use problems in the context of SV prevention. One web-based program, which used normative feedback on drinking behavior significantly reduced SV risk among women with a history of severe SV and also reduced alcohol use among college women with histories of SV and heavy episodic drinking [46]. Our own team has developed and tested college prevention programs, including a digital app (Make a Change) [47] and a web-based program for college athletes (All In) [48] that used education on consent, protective strategies, and alcohol risk reduction. These programs demonstrated reductions in SV risk factors, including violence-supportive peer norms and drinking behavior [47,48].

Prior programs offer guidance in terms of evidence-based prevention content, although none have been tested among populations seeking treatment or individuals with SUDs outside of alcohol use disorders. Although alcohol use is a commonly cited risk factor for exposure to violence, other substances are associated with IPV and SV; for example, perpetrators use illicit substances to exercise control over their partners through coercing use, encouraging the use of greater amounts, and interfering with recovery. Illicit substance use is also associated with transactional sex, stigma that leads to sexual vulnerability, and financial and relationship strain that increases risk for interpersonal violence [49-52]. Researchers describe the need for prevention programs that emphasize connections between illicit substance use and exposure to SV, as well as reducing stigma and offering supportive environments to disclose these experiences [52]. Furthermore, despite the shared risk factors and the concurrence of SV and IPV [53]*,* none of these programs integrate strategies for addressing these co-occurring forms of violence. An integrated approach to violence prevention is particularly relevant to women in SUD treatment, whose histories are characterized by multiple incidents and types of interpersonal violence [54,55].

### Theoretical Framework

**Figure 1:**
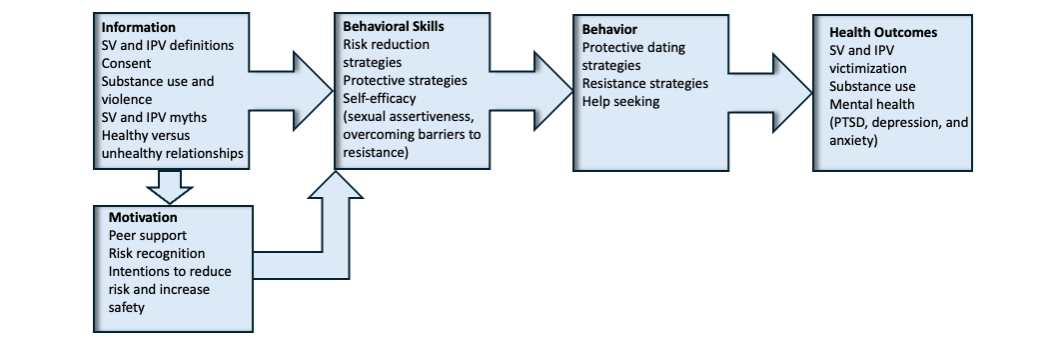
Alignment of THRIVE Program Components with the IMB Model.

The Healthy Relationships and Interpersonal Violence Education (THRIVE) program is informed by the information, motivation, behavioral skills model, which posits that three constructs influence behavior change and its consequent effects on health outcomes: (1) information and knowledge about the behavior, (2) motivation to perform the behavior, and (3) the behavioral skills necessary to perform the behavior [56]. Regarding information and knowledge, the literature on interpersonal violence indicates that inaccurate knowledge regarding how to define or label sexual assault, intentions to engage in risky behavior, overestimations of peer engagement in risky sexual behavior, acceptance of gender role stereotypes, and endorsement of rape and partner violence myths are positively associated with SV exposure risk [57-62]. Motivational factors associated with protective behaviors and exposure to SV include supportive peer norms, intentions to engage in protective behaviors, and risk recognition related to SV and IPV [63,64]. Behavioral skills and behaviors that reduce the likelihood of exposure to SV include risk recognition, coping and resistance skills for risky situations, self-efficacy to perform these skills, protective behavioral strategies (eg, sexual assertiveness and safety planning), substance use harm reduction strategies, and help seeking [65-67]. THRIVE program components align with each of these theoretical and empirical risk and protective factors as depicted in Figure 1.

### Study Aims

The study will examine the feasibility and preliminary effectiveness of THRIVE, a PSS-delivered interpersonal violence prevention program for women in substance use treatment. The first objective is to determine program acceptability and feasibility. The second objective is to determine the preliminary effectiveness of THRIVE. Proximal outcomes include violence-related knowledge and attitudes, protective sexual and dating behaviors, and help seeking. Distal outcomes include exposure to SV, substance use, and mental health. These objectives will be achieved via a single-arm trial with at least 60 women in outpatient and residential substance use treatment.

## Methods

### THRIVE Program Development

We conducted preliminary program content development following an iterative process. We first developed content based on evidence-based and theoretically informed strategies from the literature (ie, the enhanced assess, acknowledge, act model and the information, motivation, behavioral skills model), as well as our prior prevention programs with college women [47,48,68]. Second, we adapted and refined the content based on 2 rounds of interviews with 2 expert consultants, 5 health care providers at substance use treatment clinics, 5 PSSs, and 5 women in SUD treatment. The interviews assessed feasibility and acceptability, the strengths and weaknesses of the program, preferred delivery modalities, and suggestions for improvement. Participants unanimously agreed that the content would be useful and appropriate for women in SUD treatment. Participants indicated that a flexible format would be preferred in terms of offering individual versus group sessions. The women in SUD treatment noted that they would respond well and prefer a PSS over a health care or mental health provider to deliver sensitive content related to sexual assault psychoeducation and substance use.

We added components specific to this population based on feedback from the participants. These include risk reduction information focused on drug use (in contrast to the primary focus on alcohol of most prevention programs), the addition of discussions on exchanging sex for drugs, a scenario-based skills-building component, increased engagement (question-and-answer and myths vs facts exercises), and handouts summarizing key takeaways.

### Intervention

The intervention consists of two 60-minute sessions delivered in-person by PSSs in either group or individual format. Groups consist of 5 to 10 individuals. The sessions include a combination of information; interactive exercises to fuel discussion on knowledge, attitudes, and behaviors; and skills-building activities. Handouts accompany each session with key takeaways and resources. Topics include (1) definitions of SV and IPV, (2) consent, (3) healthy versus unhealthy relationships, (4) help seeking and resources for trauma-related care, (5) protective behavioral strategies, (6) safe dating, (7) alcohol and drug safety (8) sexual assertiveness, (9) overcoming barriers to protective strategies, and (10) creating a behavior change plan. Table 1 presents the intervention content. In addition to the prevention education program, PSSs screen each participant for PTSD using the Primary Care PTSD Screen for DSM-5 (PC-PTSD-5) [69]. Participants who score ≥3 (indicating a potential PTSD diagnosis) are referred to local mental health providers who provide trauma-informed care. If participants are already enrolled in mental health care, the PSS ensure that the provider is informed of the participant’s PTSD symptoms.

**Table 1:** The Healthy Relationships and Interpersonal Violence Education program content.

Sessions and topics			Content		Outcome measures		Information, motivation, behavioral skills model components
**Session 1: be in the know**							
	What is sexual assault?	Definitions and types of sexual violence Types of perpetrators		Illinois Rape Myth Acceptance Scale		Information	
	What is drug or alcohol-facilitated assault?	Definitions and types of drug- or alcohol-facilitated sexual assault How it occurs		Illinois Rape Myth Acceptance Scale		Information	
	What is consent?	What is and is not consent Enthusiastic consent		ARC3^a^ Consent Scale		Information	
	Red flags	Intimate partner violence: warning signs of unhealthy relationships		Attitudes Towards Male Dating Violence Scale		Motivation and behavioral skills	
	What is healthy?	Healthy relationships		Attitudes Towards Dating Violence Scale		Motivation and behavioral skills	
	Myths versus facts	Exercise: myths and facts about interpersonal violence		Illinois Rape Myth Acceptance Scale and Attitudes Towards Male Dating Violence Scale		Information	
	Resources	Local and national resources for interpersonal violence		Knowledge of Resources Scale		Information	
**Session 2: safety skills**							
	How to respond	How to respond if someone is pressuring or hurting you		Dating Behavior Survey		Behavioral skills	
	Alcohol and drug safety	Reducing risk when substance use is involved		Dating Behavior Survey		Information and motivation	
	Safe dates	Safe dating and protective behaviors		Barriers to Resistance Scale		Information and behavioral skills	
	Be assertive	Sexual assertiveness		Barriers to Resistance Scale		Behavioral skills	
	Overcoming barriers	Barriers to protective behaviors and how to overcome them		Resistance Responses to Sexual Aggression Scale		Motivation and behavioral skills	
	Skills building	Recognizing and responding to risk (example scenario)		Resistance Responses to Sexual Aggression Scale		Behavioral skills	
	Make a plan	Personalized behavior plan		Resistance Responses to Sexual Aggression Scale		Behavioral skills	
	Help seeking	Resources for interpersonal violence		Service Use Scale		Behavioral skills	

^a^ARC3: Administrator-Researcher Campus Climate Collaborative.

### Study Design

We developed elements for a larger clinical trial by pilot-testing a 2-session program in a single-arm trial with 60 participants. To analyze feasibility and acceptability, we will examine retention, adherence, and survey data from the trial. We also conducted exit interviews with 12 participants (10 completers and 2 noncompleters) as well as the 2 PSSs facilitating the program. To examine preliminary effectiveness, we collected self-report questionnaires at baseline, after the intervention, and at 1- and 3-month follow-ups. We assessed the proximal outcomes of knowledge, attitudes, and behaviors and the distal outcomes of exposure to SV, substance use, and mental health. Although a single-arm trial does not allow for rigorous efficacy testing, it is a first step in determining feasibility and potential efficacy. For a newly developed and exploratory intervention, it offers opportunity to identify areas for improvement and refinement to ensure a robust approach within a future large-scale, randomized controlled trial [70].

The study has been registered with ClinicalTrials.gov (NCT06608979).

### Ethical Considerations

The study was approved by the Prisma Health Institutional Review Board (2042281). Before screening potential participants for eligibility, the research coordinator obtained verbal consent for screening, which included a brief description of the study and reinforced the confidentiality of all survey responses. Interested patients who were eligible for the study after the initial screening provided verbal consent to proceed after reviewing a written informed consent document. The informed consent document reviews the possible risks and benefits of study participation and informs the participants that they may terminate the study at any time without penalty. The participants reviewed the consent document again before proceeding with each self-report survey. The surveys did not record identifying information, and participants were assigned a participant number to link survey data. To minimize social desirability bias, the study procedures emphasized with participants include confidentiality of survey responses and disclosures within the intervention sessions. This is further reinforced with the use of confidential online surveys and reliance on aggregated data for interpretation of the findings. A separate consent process was conducted for the interviews, wherein the research coordinator met with participants to review the study and asked them to provide verbal consent after reviewing the written consent document. Participants received US $30 for each of 4 assessments (a maximum of US $120). Participants completing exit interviews received an additional US $30.

### Setting

The study was conducted at 3 sites that provide substance use treatment in Greenville, South Carolina, United States. The first site, Prisma Health Addiction Medicine Center, provides care to >3000 patients with opioid use disorder per year, including medication-assisted treatment, peer recovery support, and counseling. The second site, Phoenix Center, is a comprehensive substance use treatment program with an intensive outpatient program, inpatient detoxification and rehabilitation programs, an outpatient buprenorphine program, and a residential program for pregnant women and mothers (Serenity Place). Phoenix Center serves >2000 individuals per year. PSSs are contracted to work with the patients at Phoenix Center. The third site, Magdalene Clinic at Prisma Health, offers prenatal care, peer support services, and counseling to pregnant persons with SUDs. Magdalene Clinic serves >100 individuals per year and works closely with Prisma Health Addiction Medicine Center and Phoenix Center.

### Participants and Procedure

#### Participants

We recruited and enrolled 92 women (71 completers) in substance use treatment for a single-arm trial of THRIVE. The inclusion criteria include (1) adults (aged ≥18 y), (2) identifying as women, (3) currently enrolled in substance use treatment (behavioral or pharmacological), and (4) willing to accept random assignment to a waitlist (usual care) or THRIVE. The exclusion criteria include (1) having a severe medical or psychiatric disability that could impair ability to perform study-related activities (determined by their substance use treatment provider), (2) unable to independently read and comprehend the consent form or other study materials, and (3) unable to read and speak English. A total of 10 to 20 women as well as the 2 PSSs were selected to complete exit interviews. The inclusion criteria for the interviews included (1) women who completed either the baseline survey only (intervention noncompleters) or the full THRIVE intervention (intervention completers); and (2) for the PSSs, those who delivered the THRIVE intervention.

#### Recruitment

Participants were recruited via referral from clinicians and research coordinators at the 3 recruitment sites, using flyers, email, and verbal communication. Recruitment occurred during each clinic’s operating hours (8:30 AM-5 PM). Recruitment for the pilot trial remained open until at least 60 participants completed the intervention. For the interviews, women who completed either the baseline assessment session only or the full THRIVE intervention were randomly selected and recruited via telephone or email by the research coordinator. Recruitment for interviews remained open until data saturation was achieved, at 12 participants [71].

#### Screening

The research coordinator enrolled potential participants from the Prisma Health Addiction Medicine Center, Phoenix Center, and Magdalene Clinic by contacting patients who were referred by clinicians and staff at each of these facilities. Potentially eligible participants were given a flyer about the study and asked whether they were interested in considering participation. Potential participants who expressed interest in learning about our study were referred to the research coordinator, who described the study to the participants and initiated the consent process.

#### Assessment Plan

Research assessments consisted of 20- to 30-minute online self-report questionnaires at 4 assessment points: baseline, after the intervention, and 1- and 3-month follow-ups. Ten completers and 2 noncompleters from the intervention group were selected for the postintervention exit interviews. We also conducted interviews with the 2 PSSs who are facilitating the program. Participants attended baseline, intervention, and postintervention visits in person at 1 of the 3 study sites. The research coordinator or another research team member attended all baseline and postintervention visits (immediately before and after the THRIVE sessions) in person to collect online survey data using a tablet PC. Qualtrics software (Qualtrics International Inc) [72] was used to administer the online surveys and extract the data. Follow-up data were collected via online surveys delivered to participants via SMS text message or email. For the interviews, participants were provided with a copy of the THRIVE prevention program content to review, either via email or in hard copy. Participants then completed a 30-minute semistructured interview assessing program acceptability, feasibility, strengths, weaknesses, and areas for revision or improvement, as well as barriers and facilitators to participation, engagement, and implementation. A member of the research team conducted the interviews via teleconference for the PSSs and in person for the women in treatment.

#### Retention Plan

Beyond the incentive structure for study participation, implemented additional strategies to ensure high retention rates. First, we asked for ≥2 alternate contacts at baseline so that we could continue to reach participants during follow-up. Written consent was also obtained to contact other persons for locating the participant for follow-up. The research coordinator contacted all participants at least once between follow-up assessments to maintain a connection and updated contact information. Participants received at least 3 email or SMS text message reminders to complete surveys. The research coordinator made at least 20 attempts to contact participants who did not respond or complete assessments before considering them lost to follow-up. The research coordinator attempted to make regular contact with participants to collect data at each assessment interval, regardless of whether they attended the intervention. The research coordinator also worked with health care providers to determine when eligible participants had appointments to better facilitate scheduling assessment sessions around already scheduled health care appointment times.

Research team members attended staff meetings at each recruitment site to update the sites on enrollment and retention status and to work with the sites on overcoming barriers to recruitment and retention. A member of the research team was present on site throughout active recruiting periods to connect with health care providers, screen eligible participants, and meet with potential participants around the time of their health care appointments.

#### Measures

##### Overview

Table 2 presents a list of outcomes and study measures.

**Table 2:** Pilot trial outcomes and measures.

Outcomes and measures				Baseline		Sessions 1-2		After the intervention		1-month follow-up		3-month follow-up		Information, motivation, behavioral skills model components		Health outcome
**Acceptability and feasibility**																
	**Feasibility**															
		Recruitment and retention: enrollment and attendance tracking					✓				✓					
		Percentage of assessments completed					✓				✓					
	**Adherence**															
		THRIVE^a^ adherence scale (rating of PSS^b^ fidelity to protocol)			✓											
	**Acceptability and engagement**															
		PSS interviews							✓							
		Exit interviews							✓							
		Satisfaction and engagement scale					✓		✓							
		Qualitative survey items: feedback on protocol					✓		✓							
**Proximal outcomes: knowledge, attitudes, and behaviors**																
	**Sexual violence attitudes**															
		Illinois Rape Myth Acceptance Scale–Short Form [73]	✓				✓		✓		✓		✓^c^			
	**Partner violence attitudes**															
		Attitudes Towards Dating Violence Scale [74]	✓				✓		✓		✓		✓^c^			
	**Knowledge of consent**															
		ARC3^d^ Consent Scale [75]	✓				✓		✓		✓		✓^c^			
	**Sexual self-efficacy**															
		Barriers to Resistance Scale [67]	✓				✓		✓		✓		✓^e^			
	**Behavioral intentions**															
		Dating Behavior Survey [76]	✓				✓		✓		✓		✓^f^			
		Resistance Responses to Sexual Aggression Scale [77]	✓				✓		✓		✓		✓^f^			
	**Knowledge of resources**															
		Knowledge of Resources Scale (locally adapted) [75]	✓				✓		✓		✓		✓^c^			
	**Help seeking (trauma-related service use)**															
		Frequency of use of behavioral health services and local and national resources	✓						✓		✓		✓^e^			
**Primary distal outcomes: exposure to violence**																
	**Exposure to sexual violence**															
		Sexual Experiences Survey–Short Form [78]	✓						✓		✓				✓	
	**Exposure to intimate partner violence**															
		Revised Conflict Tactics Scale–Short Form [79]	✓						✓		✓				✓	
**Secondary distal outcomes: substance use and mental health**																
	**Substance use**															
		CAGE-AID^g^ [80]	✓				✓		✓		✓		✓^e^			
	**PTSD** ^h^															
		PC-PTSD-5^i^ [69]	✓				✓		✓		✓				✓	
	**Depression**															
		PHQ-2^j^ [81]	✓				✓		✓		✓				✓	

^a^THRIVE: The Healthy Relationships and Interpersonal Violence Education program.

^b^PSS: peer support specialist.

^c^Information.

^d^ARC3: Administrator-Researcher Campus Climate Collaborative.

^e^Behavior skills.

^f^Motivation.

^g^CAGE-AID: CAGE Adapted to Include Drugs.

^h^PTSD: posttraumatic stress disorder.

^i^PC-PTSD-5: Primary Care PTSD Screen for DSM-5.

^j^PHQ-2: Patient Health Questionnaire-2.

##### Demographics

A demographics questionnaire assessed gender identity, race and ethnicity, and age. A participant log recorded recruitment site and intervention format.

##### Feasibility and Acceptability

The data sources are presented in Textbox 1.

**Data sources.**

- Recruitment and retention: study logs recorded enrollment, attendance, retention rates, and the percentage of assessments completed.
- Adherence scales: completed by research team members for 50% of the sessions, these scales assess peer support specialist fidelity to the protocol.
- Satisfaction and engagement scale: 4 Likert scale survey items from our prior study (eg, “How engaging did you find the program?”) assess satisfaction and engagement.
- Qualitative feedback: 2 items on the self-report surveys elicited areas for improvement and strengths of the protocol (eg, “What did you like about the program?” and “How could the program be improved?”).
- Interviews: semistructured interviews assessed program feasibility, acceptability, strengths, and weaknesses (eg, feasibility of attending appointments, acceptability of the study location, satisfaction with the research team, and intervention format and modality). The interviews also assessed alliance with peer support specialists and suggestions for improvement (eg, “How appropriate does the program seem for women who are in substance use treatment?” “How did you feel about your interactions with the peer support specialist who delivered the program?” “What barriers or difficulties did you experience in terms of participating in the study?”).

##### Knowledge, Attitudes, and Behaviors

Proximal outcomes were assessed via the self-report surveys presented in Textbox 2 [67,73-77].

**Proximal outcomes and measures.**

- The Illinois Rape Myth Acceptance Scale [73] is a 20-item scale assessing acceptance of rape-supportive beliefs and norms (eg, “If a woman was raped while she was drunk, she is responsible for what happened”), with total scores ranging from 20 to 140. Higher scores indicate greater rape myth acceptance.
- The Attitudes Towards Male Dating Violence Scale [74] consists of 15 items assessing acceptance of dating and partner violence myths, including stereotypical gender roles and acceptance of partner violence, (eg, “A girl should always do what her boyfriend or intimate partner tells her to do”), with scores ranging from 15 to 105. Higher scores represent greater acceptance of dating violence myths.
- The Administrator-Researcher Campus Climate Collaborative Consent Scale [75], a 7-item scale ranging from 7 to 35, assesses understanding of consent in sexual situations (eg, “If you and your partner are both drunk, you don’t have to worry about consent”), with higher scores reflecting a lower understanding of consent.
- The Barriers to Resistance Scale [67] is a 13-item scale that assesses sexual self-efficacy, sexual assertiveness, and perceived barriers to resisting in sexually coercive situations, including substance use (eg, “I might be too intoxicated to think through a plan to get out of the situation”). Total scores range from 13 to 52, with higher scores indicating greater perceived barriers.
- The Dating Behavior Survey [76], a 15-item scale, with scores ranging from 7 to 105, assesses intentions to engage in risky and protective behaviors, such as using substances on a date, arranging for transportation, and making safety plans (eg, “On the first few dates that we have, I will allow my partner to plan what we do”). Higher scores reflect greater intentions to engage in these behaviors.
- The Resistance Responses to Sexual Aggression Scale [77] is a 16-item scale assessing the perceived likelihood of engaging in resistance responses in sexually aggressive situations, including verbal and physical strategies (eg, “Tell them I like them but I’m not ready”). Total scores range from 16 to 112, with higher scores suggesting a greater perceived likelihood of using resistance responses.
- The Knowledge of Resources Scale [75] was adapted to local context to assess familiarity with local and national resources for trauma-focused care (resulting in 13 items), with total scores ranging from 13 to 65. Higher scores indicate a greater awareness of resources for trauma-focused care.
- Trauma-related service use was assessed as the past-month frequency of accessing a list of local and national resources related to interpersonal violence (13 items). The outcome measures were self-report scales that were summed to create mean and total scores.

##### Exposure to SV and IPV

Outcomes regarding exposure to SV and IPV were assessed using the Sexual Experiences Survey–Short Form [78] and the Revised Conflict Tactics Scale [79], respectively.

The Sexual Experiences Survey–Short Form is a 7-item scale that assesses self-reported experiences of various forms of exposure to SV (unwanted sexual contact, sexual coercion, attempted rape, and rape) using several tactics (verbal, physical, and drug or alcohol facilitation).

The Revised Conflict Tactics Scale, a 20-item measure of IPV, assesses psychological, physical, and sexual experiences of IPV in the context of a marital, dating, or cohabitating relationship, with total scores ranging from 20 to 100. Higher scores indicate greater frequency of experiences of exposure to IPV. Exposure to IPV will be scored as a dichotomous outcome and will be measured across 2 time frames: lifetime and past 3 months.

##### Substance Use and Mental Health

A secondary set of distal outcomes was assessed with the CAGE Adapted to Include Drugs Questionnaire [80], the PC-PTSD-5 [69], and the Patient Health Questionnaire-2 [81].

The CAGE Adapted to Include Drugs Questionnaire is a 4-item conjoint screening questionnaire for alcohol and other drug use (eg, “Have you felt the need to cut down on your drinking or drug use?). Scores range from 0 to 4, with higher scores indicating greater risk of problematic substance use.

The PC-PTSD-5 is a 5-item scale that screens for Diagnostic and Statistical Manual of Mental Disorders, Fifth Edition, PTSD criteria, with total scores ranging from 0 to 5. Higher scores indicate a greater likelihood of probable PTSD.

The Patient Health Questionnaire-2 is a 2-item scale that screens for Diagnostic and Statistical Manual of Mental Disorders, Fifth Edition depression criteria. Total scores range from 2 to 8, with higher scores reflecting greater depressive symptoms.

The distal outcome measures are self-report scales that will be summed to create total scores.

#### Quality Assurance and Implementation

Two female PSSs were hired for the study. Both were trained as certified PSSs and certified assertive community engagement specialists through the National Association of Alcohol and Drug Abuse Counselors program. They are required to be at least 1 year in recovery from addiction, as defined by SAMHSA. Certified PSSs in South Carolina must have 2 recommendation letters from employers or supervisors attesting to 1 year of recovery to receive certification [82]. Experience with interpersonal violence or recovery from trauma is not specified as a requirement to perform as a PSS in this capacity. PSSs received at least 10 hours of training from the principal investigator (PI), using the PSS training manual and the THRIVE intervention protocol developed in our prior research. As part of the training, PSSs conducted at least 2 mock sessions under observation by the PI. PSSs also met weekly with the PI, a licensed clinical psychologist, for supervision once they began delivering the program. Although not a criterion for the study, PSSs may have a history of interpersonal violence, which was assessed and addressed by the PI to ensure that their own mental health concerns were adequately managed and monitored. If the PSSs had trauma symptoms interfering with the delivery of the intervention that could not be adequately managed via clinical supervision, they would have been referred for mental health treatment and could be removed from their role in the study. PSSs were trained in recognizing adverse events and signs of distress and referring participants to mental health and medical professionals to address these concerns as they arise. Intervention fidelity was monitored with a checklist developed by the research team to assess the completion of each THRIVE component. After achieving reliability with at least one other research team member during initial training sessions, one member of the research team attended and rated each of the sessions using the intervention checklist.

#### Managing Participant Distress

Facilitators were trained in recognizing signs of distress, intoxication, and overdose. Facilitators were instructed to refer participants in distress, including those expressing suicidal or homicidal ideation, to emergency medical personnel on site. For other mental health concerns, including PTSD and trauma-related symptoms arising during the intervention, facilitators were instructed to refer to their current provider or to a social worker at the Prisma Health Addiction Medicine Center. If a participant appeared to be intoxicated, the facilitator was instructed to contact the patient’s provider or recovery program nurse, ensure that a referral is completed, and also ensure that the participant had transportation and was not driving. Participants who were intoxicated would not be allowed to participate in study sessions at that time and would be asked to reschedule. If a participant showed signs of overdose, the facilitator would call 911 or emergency medical services and ask recovery program staff for assistance. They could also escort the participant to the emergency department. If a participant was agitated, the facilitator was instructed to contact security. All participants were given handouts with local and national resources for counseling, shelter, crisis management, and related services. In addition, participants were made aware that the PSSs are available to assist with follow-up needs at the conclusion of the intervention. Site-specific procedures and contact persons were outlined in the facilitator training manuals.

### Analysis

#### Feasibility and Acceptability

Descriptive statistics will be used to describe recruitment and retention rates and to calculate means on survey items assessing feasibility and acceptability. A benchmark of ≥75% of the *agree* and *strongly agree* (equivalent to an average score of ≥4) responses will be used [83,84]. For the satisfaction and engagement scale, qualitative survey items will be coded by 2 research team members. Interviews will be recorded, transcribed, and uploaded to NVivo (Lumivero) [85] for thematic analysis [86]. The research team will conduct a line-by-line reading of transcripts to identify inductive codes that emerge from the data. The team will then collaborate to develop a codebook that includes operationalized definitions of each of these inductive codes as they apply to the topics addressed in the interviews (ie, feasibility, acceptability, strengths, weaknesses, areas for improvement, and barriers and facilitators to implementation). A study investigator and the research coordinator will code a subset of the data, then convene to resolve discrepancies and revise codes until consensus is achieved on the codebook. Two research team members will then code the entire dataset and meet to achieve consensus on the final codes. The results from these analyses will be used to inform further refinement of the THRIVE protocol before proceeding to a larger randomized controlled trial.

#### Preliminary Effectiveness

##### Analytic Sample

The primary analytic sample will include all participants who completed the baseline survey; this will serve as the intention-to-treat sample. We will also conduct modified intention-to-treat analyses including only participants who attended 1 THRIVE session.

##### Missing Data

Every effort was made to minimize missing primary outcome data. However, the primary analytic strategy relies on mixed effects models, which is unbiased under missing-at-random assumptions and is the most frequently accepted approach in longitudinal data analysis [87]. Characteristics of patients who are lost to follow-up will be compared to those who complete the study to assess the degree of any selection bias due to attrition. We will complete a sensitivity analysis to compute indices of sensitivity to the nonignorability of missing data. If these analyses indicate evidence that nonignorability will impact model parameter estimation, we will apply fully conditional specification multiple imputation methods, which are applicable to nonignorable missing data [87,88].

#### Statistical Analyses

To test the effects of THRIVE on primary and secondary outcomes, linear mixed effects model for continuous outcomes and generalized linear mixed effects models for binary outcomes will be used, with subject-specific random intercepts to account for the correlations of longitudinal outcomes measured after the intervention (week 2) and at 1-month and 3-month follow-ups. Covariates, such as recruitment sites, any demographic variables correlated with primary outcomes, type of substance use, and intervention format, will be included as fixed effects in all analysis models. The primary knowledge, attitude, and behavior outcomes will be continuous outcomes, and secondary outcomes such as experience of SV or IPV will be binary outcomes. In addition, with this model, we will also identify postbaseline time points where changes in outcomes from baseline are significant.

## Results

Recruitment, enrollment, and data collection from the 3 sites was completed between June 2024 and March 2025. Of the 92 women recruited and enrolled, 71 (77%) completed the intervention and postintervention assessments, 58 (63%) completed the 1-month follow-up assessments, and 44 (48%) completed the 3-month follow-up assessments. Ten completers, 2 noncompleters, and 2 PSS completed exit interviews. Of the 92 enrolled participants, 45 (50%) were from the Prisma Health Addiction Medicine Center, 42 (47%) from Phoenix Center, and 3 (3%) from Magdalene Clinic. Of the 92 participants, 1 (1%) was American Indian or Alaska Native, 4 (4%) were Black, 1 (1%) were Hispanic, 79 (86%) were White, 2 (2%) identified as multiracial, and 3 (3%) identified as “other.” Racial and ethnic distribution is representative of the patient populations at the recruitment sites. The participants’ mean age was 35 (SD 9.87) years. The most commonly reported substances used included tobacco (79/92, 86%), cannabis (58/92, 63%), alcohol (55/92, 61%), amphetamine-type stimulants (58/92, 63%), and opioids (50/92, 54%; Table 3).

**Table 3:** Types of substances used by enrolled participants (N=92).

Substance types	Participants, n (%)
Tobacco	79 (86)
Alcohol	55 (61)
Cannabis	58 (63)
Opioids (heroin, fentanyl, etc)	50 (54)
Amphetamine-type stimulants	58 (63)
Inhalants	19 (24)
Sedatives	47 (51)
Hallucinogens	32 (35)
Cocaine	44 (48)

## Discussion

### Summary

This study evaluated an interpersonal violence prevention program for women with SUDs to fill a vacuum in programs with demonstrated efficacy for this population. This will be the first study to test an evidence-based SV and IPV prevention program for women in substance use treatment. It will address the nexus of 2 significant public health problems: interpersonal violence and SUDs. Although a majority of women in substance use treatment have a history of SV or IPV, a history of interpersonal violence and risk for repeated exposure to interpersonal violence are rarely addressed in substance use treatment settings.

Both a history of violence and substance use place women at risk for cumulative exposure to violence and adverse outcomes, including SUDs, PTSD, depression, and physical health problems. For women in substance use treatment, exposure to SV and IPV can lead to escalation of substance use and inability to achieve recovery [11]. However, women face barriers to care in seeking trauma-focused services, and there is a lack of available evidence-based violence prevention programs within substance use treatment settings. Therefore, there is a significant need for research to develop and test violence prevention services that could mitigate the sequelae of initial and repeated exposure to interpersonal violence among women in substance use treatment.

An additional gap in the literature that will be addressed by the THRIVE program is the lack of integrated prevention programming for SV and IPV, despite their shared risk factors. These forms of violence share risk factors such as exposure to childhood adversity, stereotypical gender role conformity, and substance use [53]. An intervention that is both pragmatic and designed to achieve maximum impact will address multiple forms of interpersonal violence simultaneously. The THRIVE program is one of only a few programs to adopt an integrated approach to these 2 co-occurring forms of violence [53,89].

THRIVE will also be the only known program to address the use of illicit substances as a risk reduction strategy for violence prevention. Prior prevention programs have singularly focused on alcohol use [46]. However, our data from the program development phase suggested the need to address unique concerns around illicit substance use, such as vulnerability during withdrawal, coercion by dealers, and exchange of sex for drugs.

Furthermore, THRIVE will be the first program to engage PSSs as violence prevention educators. Consistent with recommended models of trauma-informed care in substance use treatment settings [90], we will incorporate trained PSSs as educators who can lift stigma, offer empathy and trust, reduce barriers to engagement, and fill gaps in mental health service provision. While PSSs have delivered prevention programming in the mental health sector [91], this will be the first known study to develop and test a PSS-delivered violence prevention program. In addition, training PSSs in health promotion skills related to violence and substance use will provide them with professional skills and confidence to work with their clientele. A PSS violence prevention model also offers scalable and cost-effective solutions for workforce development and the expansion of mental health services.

Finally, THRIVE places unique emphasis on help seeking and overcoming barriers to care as violence prevention strategies. Encouraging help seeking will promote access to resources that could mitigate mental health conditions and reduce adversity related to exposure to interpersonal violence as a means of both health promotion and risk reduction. Our preliminary research indicates that participants view this content as both necessary and missing from existing prevention programs [48]. One benefit of using PSSs as facilitators is that they are trained and capable of both modeling appropriate resource use and providing warm handoffs to other services.

### Conclusions

Upon successful completion of this study, we expect to demonstrate the feasibility, acceptability, and preliminary effectiveness of THRIVE. This program will address gaps in the evidence base by emphasizing the critical intersection of violence and substance use, expanding effective strategies to a population considered high risk, and implementing a novel peer-delivered format to overcome barriers to care. The limitations of this approach include the use of a single-arm trial, underrepresentation of minoritized populations at our recruitment sites, and flexibility in program format that introduces some variability and could limit generalizability. If preliminary feasibility and efficacy are established, we will use the study findings to design future trials that minimize these limitations by using randomized controlled designs, oversampling underrepresented groups, and standardizing the delivery format. Our long-term goal is to develop an accessible, scalable, and efficacious prevention program that reduces the incidence of exposure to SV and IPV, substance use, and violence-related mental health disorders for women in substance use treatment. Ultimately, reducing exposure to SV and IPV is expected to reduce substance use escalation as well as sustain recovery from SUDs over the long term.

## Abbreviations

IPV: intimate partner violence
PC-PTSD-5: Primary Care PTSD Screen for DSM-5
PI: principal investigator
PSS: peer support specialist
PTSD: posttraumatic stress disorder
SAMHSA: Substance Abuse and Mental Health Services Administration
SUD: substance use disorder
SV: sexual violence
THRIVE: The Healthy Relationships and Interpersonal Violence Education

## References

[1] Hines DA (2007). Predictors of sexual coercion against women and men: a multilevel, multinational study of university students. Arch Sex Behav.

[2] Green JG, McLaughlin KA, Berglund PA, Gruber MJ, Sampson NA, Zaslavsky AM, Kessler RC (2010). Childhood adversities and adult psychiatric disorders in the national comorbidity survey replication I: associations with first onset of DSM-IV disorders. Arch Gen Psychiatry.

[3] Pirard S, Sharon E, Kang SK, Angarita GA, Gastfriend DR (2005). Prevalence of physical and sexual abuse among substance abuse patients and impact on treatment outcomes. Drug Alcohol Depend.

[4] Krug EG, Mercy JA, Dahlberg LL, Zwi AB (2002). The world report on violence and health. Lancet.

[5] Breiding MJ, Smith SG, Basile KC, Walters ML, Chen J, Merrick MT (2014). Prevalence and characteristics of sexual violence, stalking, and intimate partner violence victimization--national intimate partner and sexual violence survey, United States, 2011. MMWR Surveill Summ.

[6] Tjaden PG, Thoennes N (2006). Extent, nature, and consequences of rape victimization: findings from the national violence against women survey. Office of Justice Programs, U.S. Department of Justice.

[7] Breiding M, Basile KC, Smith SG, Black MC, Mahendra RR (2015). Intimate partner violence surveillance: uniform definitions and recommended data elements. Version 2.0. National Center for Injury Prevention and Control (U.S.).

[8] Kessler RC, Aguilar-Gaxiola S, Alonso J, Benjet C, Bromet EJ, Cardoso G, Degenhardt L, de Girolamo G, Dinolova RV, Ferry F, Florescu S, Gureje O, Haro JM, Huang Y, Karam EG, Kawakami N, Lee S, Lepine J, Levinson D, Navarro-Mateu F, Pennell B, Piazza M, Posada-Villa J, Scott KM, Stein DJ, Ten Have M, Torres Y, Viana MC, Petukhova MV, Sampson NA, Zaslavsky AM, Koenen KC (2017). Trauma and PTSD in the WHO world mental health surveys. Eur J Psychotraumatol.

[9] Schnurr PP, Green BL (2004). Understanding relationships among trauma, post-tramatic stress disorder, and health outcomes. Adv Mind Body Med.

[10] Zinzow HM, Resnick HS, McCauley JL, Amstadter AB, Ruggiero KJ, Kilpatrick DG (2012). Prevalence and risk of psychiatric disorders as a function of variant rape histories: results from a national survey of women. Soc Psychiatry Psychiatr Epidemiol.

[11] Bishop LS, Benz MB, Palm Reed KM (2017). The impact of trauma experiences on posttraumatic stress disorder and substance use disorder symptom severity in a treatment-seeking sample. Prof Psychol Res Pr.

[12] Clark HW, Masson CL, Delucchi KL, Hall SM, Sees KL (2001). Violent traumatic events and drug abuse severity. J Subst Abuse Treat.

[13] Farley M, Golding JM, Young G, Mulligan M, Minkoff JR (2004). Trauma history and relapse probability among patients seeking substance abuse treatment. J Subst Abuse Treat.

[14] Ruback RB, Clark VA, Warner C (2014). Why are crime victims at risk of being victimized again? Substance use, depression, and offending as mediators of the victimization-revictimization link. J Interpers Violence.

[15] Walsh K, Resnick HS, Danielson CK, McCauley JL, Saunders BE, Kilpatrick DG (2014). Patterns of drug and alcohol use associated with lifetime sexual revictimization and current posttraumatic stress disorder among three national samples of adolescent, college, and household-residing women. Addict Behav.

[16] Sells DJ, Rowe M, Fisk D, Davidson L (2003). Violent victimization of persons with co-occurring psychiatric and substance use disorders. Psychiatr Serv.

[17] Ducharme LJ, Knudsen HK, Roman PM (2006). Availability of integrated care for co-occurring substance abuse and psychiatric conditions. Community Ment Health J.

[18] Substance use disorder treatment options. Substance Abuse and Mental Health Services Administration.

[19] (2014). Trauma-informed care in behavioral health services. Center for Substance Abuse Treatment (US), US Department of Health and Human Services.

[20] Capezza NM, Najavits LM (2012). Rates of trauma-informed counseling at substance abuse treatment facilities: reports from over 10,000 programs. Psychiatr Serv.

[21] Spivak S, Spivak A, Decker MR, Cullen B, Yao M, Mojtabai R (2022). Availability of trauma-specific services in US substance use disorder and other mental health treatment facilities: 2015-2019. Psychiatr Q.

[22] Killeen TK, Back SE, Brady KT (2015). Implementation of integrated therapies for comorbid post-traumatic stress disorder and substance use disorders in community substance abuse treatment programs. Drug Alcohol Rev.

[23] Zinzow HM, Littleton H, Muscari E, Sall K (2021). Barriers to formal help-seeking following sexual violence: review from within an ecological systems framework. Vict Offender.

[24] Robinson SR, Ravi K, Voth Schrag RJ (2021). A systematic review of barriers to formal help seeking for adult survivors of IPV in the United States, 2005-2019. Trauma Violence Abuse.

[25] Zweig JM, Schlichter KA, Burt MR (2002). Assisting women victims of violence who experience multiple barriers to services. Violence Against Women.

[26] Coombs NC, Meriwether WE, Caringi J, Newcomer SR (2021). Barriers to healthcare access among U.S. adults with mental health challenges: a population-based study. SSM Popul Health.

[27] Singh V, Kumar A, Gupta S (2022). Mental health prevention and promotion-a narrative review. Front Psychiatry.

[28] (2009). What are peer recovery support services?. Substance Abuse and Mental Health Services Administration.

[29] National model standards for peer support certification. Substance Abuse and Mental Health Services Administration.

[30] Stratford AC, Halpin M, Phillips K, Skerritt F, Beales A, Cheng V, Hammond M, O'Hagan M, Loreto C, Tiengtom K, Kobe B, Harrington S, Fisher D, Davidson L (2019). The growth of peer support: an international charter. J Ment Health.

[31] Mead S, Hilton D, Curtis L (2001). Peer support: a theoretical perspective. Psychiatr Rehabil J.

[32] Earnshaw VA (2020). Stigma and substance use disorders: a clinical, research, and advocacy agenda. Am Psychol.

[33] Waterman EA, Banyard VL, Edwards KM, Mauer VA (2022). Youth perceptions of prevention norms and peer violence perpetration and victimization: a prospective analysis. Aggress Behav.

[34] Hackman CL, Witte T, Greenband M (2017). Social norms for sexual violence perpetration in college. J Aggress Confl Peace Res.

[35] Schlichthorst M, Ozols I, Reifels L, Morgan A (2020). Lived experience peer support programs for suicide prevention: a systematic scoping review. Int J Ment Health Syst.

[36] Fuhr DC, Salisbury TT, De Silva MJ, Atif N, van Ginneken N, Rahman A, Patel V (2014). Effectiveness of peer-delivered interventions for severe mental illness and depression on clinical and psychosocial outcomes: a systematic review and meta-analysis. Soc Psychiatry Psychiatr Epidemiol.

[37] Edwards KM, Jones LM, Mitchell KJ, Hagler MA, Roberts LT (2016). Building on youth’s strengths: a call to include adolescents in developing, implementing, and evaluating violence prevention programs. Psychol Violence.

[38] An S, Welch-Brewer C, Tadese H (2024). Scoping review of intimate partner violence prevention programs for undergraduate college students. Trauma Violence Abuse.

[39] Kettrey HH, Thompson MP, Marx RA, Davis AJ (2023). Effects of campus sexual assault prevention programs on attitudes and behaviors among American college students: a systematic review and meta-analysis. J Adolesc Health.

[40] Graham LM, Embry V, Young B, Macy RJ, Moracco KE, Reyes HL, Martin SL (2021). Evaluations of prevention programs for sexual, dating, and intimate partner violence for boys and men: a systematic review. Trauma Violence Abuse.

[41] Koss MP, Swartout KM, Lopez EC, Lamade RV, Anderson EJ, Brennan CL, Prentky RA (2022). The scope of rape victimization and perpetration among national samples of college students across 30 years. J Interpers Violence.

[42] Porat R, Gantman A, Green SA, Pezzuto J, Paluck EL (2024). Preventing sexual violence: a behavioral problem without a behaviorally informed solution. Psychol Sci Public Interest.

[43] Senn CY, Eliasziw M, Hobden KL, Newby-Clark IR, Barata PC, Radtke HL, Thurston WE (2017). Secondary and 2-year outcomes of a sexual assault resistance program for university women. Psychol Women Q.

[44] Senn CY, Eliasziw M, Barata PC, Thurston WE, Newby-Clark IR, Radtke HL, Hobden KL (2015). Efficacy of a sexual assault resistance program for university women. N Engl J Med.

[45] Senn CY, Eliasziw M, Barata PC, Thurston WE, Newby-Clark IR, Radtke HL, Hobden KL, SARE study team (2013). Sexual assault resistance education for university women: study protocol for a randomized controlled trial (SARE trial). BMC Womens Health.

[46] Gilmore AK, Lewis MA, George WH (2015). A randomized controlled trial targeting alcohol use and sexual assault risk among college women at high risk for victimization. Behav Res Ther.

[47] Orchowski LM, Zinzow HM, Thompson MP, Wood S (2023). Open pilot trial of an interactive digital application for campus sexual violence prevention. J Community Psychol.

[48] Thompson MP, Zinzow HM, Kingree JB, Pollard LE, Goree J, Hudson-Flege M, Honnen NG (2021). Pilot trial of an online sexual violence prevention program for college athletes. Psychol Violence.

[49] Warshaw C, Lyon E, Bland P, Phillips H, Hooper M (2014). Mental health and substance use coercion surveys report. National Center on Domestic Violence, Trauma, and Mental Health.

[50] Gilbert L, El-Bassel N, Rajah V, Foleno A, Frye V (2001). Linking drug-related activities with experiences of partner violence: a focus group study of women in methadone treatment. Violence Vict.

[51] Jiwatram-Negron T, Peitzmeier S, Meinhart M, Vasiliou N, Nikitin D, Gilbert L (2022). Associations between transactional sex and intimate and non-intimate partner violence: findings from project WINGS of Hope. J Fam Viol.

[52] Jessell L, Mateu-Gelabert P, Guarino H, Vakharia SP, Syckes C, Goodbody E, Ruggles KV, Friedman S (2017). Sexual violence in the context of drug use among young adult opioid users in New York City. J Interpers Violence.

[53] Abramsky T, Watts CH, Garcia-Moreno C, Devries K, Kiss L, Ellsberg M, Jansen HA, Heise L (2011). What factors are associated with recent intimate partner violence? Findings from the WHO multi-country study on women's health and domestic violence. BMC Public Health.

[54] Schacht RL, Wenzel KR, Meyer LE, Mette M, Mallik-Kane K, Rabalais A, Berg SK, Fishman M (2023). A pilot test of written exposure therapy for PTSD in residential substance use treatment. Am J Addict.

[55] Dworkin ER, Mota NP, Schumacher JA, Vinci C, Coffey SF (2017). The unique associations of sexual assault and intimate partner violence with PTSD symptom clusters in a traumatized substance-abusing sample. Psychol Trauma.

[56] Fisher WA, Fisher JD, Harman J, Suls J, Wallston KA (2003). The information-motivation-behavioral skills model: a general social psychological approach to understanding and promoting health behavior. Social Psychological Foundations of Health and Illness.

[57] Franklin CA (2012). Anticipating intimacy or sexual victimization? Danger cue recognition and delayed behavioral responses to a sexually risky scenario. Fem Criminol.

[58] Foshee VA, Benefield TS, Ennett ST, Bauman KE, Suchindran C (2004). Longitudinal predictors of serious physical and sexual dating violence victimization during adolescence. Prev Med.

[59] Combs-Lane AM, Smith DW (2002). Risk of sexual victimization in college women: the role of behavioral intentions and risk-taking behaviors. J Interpers Violence.

[60] Littleton H, Axsom D, Grills-Taquechel A (2009). Sexual assault victims' acknowledgment status and revictimization risk. Psychol Women Q.

[61] Lewis MA, Litt DM, Cronce JM, Blayney JA, Gilmore AK (2014). Underestimating protection and overestimating risk: examining descriptive normative perceptions and their association with drinking and sexual behaviors. J Sex Res.

[62] Ullman S, Najdowski C, White JW, Koss MP, Kazdin AE (2011). Vulnerability and protective factors for sexual assault. Violence Against Women and Children, Vol 1: Mapping the Terrain.

[63] Orchowski LM, Untied AS, Gidycz CA (2012). Reducing risk for sexual victimization: an analysis of the perceived socioemotional consequences of self-protective behaviors. J Interpers Violence.

[64] Zaso MJ, Livingston JA, Shaw RJ, Colder CR, Read JP (2024). Self-Protection in the social context: a daily-level examination of young adult women's perceived need for and engagement in sexual assault protective behavioral strategies. Psychol Violence.

[65] Kelley EL, Orchowski LM, Gidycz CA (2016). Sexual victimization among college women: role of sexual assertiveness and resistance variables. Psychol Violence.

[66] Rozee PD, Koss MP (2001). Rape: a century of resistance. Psychol Women Q.

[67] Norris J, Nurius PS, Dimeff LA (1996). Through Her Eyes: factors affecting women's perception of and resestance to acquaintance sexual aggression threat. Psychol Women Q.

[68] Senn CY, Eliasziw M, Hobden KL, Barata PC, Radtke HL, Thurston WE, Newby-Clark IR (2020). Testing a model of how a sexual assault resistance education program for women reduces sexual assaults. Psychol Women Q.

[69] Prins A, Bovin MJ, Smolenski DJ, Marx BP, Kimerling R, Jenkins-Guarnieri MA, Kaloupek DG, Schnurr PP, Kaiser AP, Leyva YE, Tiet QQ (2016). The primary care PTSD screen for DSM-5 (PC-PTSD-5): development and evaluation within a veteran primary care sample. J Gen Intern Med.

[70] Wang M, Ma H, Shi Y, Ni H, Qin C, Ji C (2024). Single-arm clinical trials: design, ethics, principles. BMJ Support Palliat Care.

[71] Ahmed SK (2025). Sample size for saturation in qualitative research: debates, definitions, and strategies. J Med Surg Public Health.

[72] (2005). Qualtrics survey software. Qualtrics.

[73] Payne DL, Lonsway KA, Fitzgerald LF (1999). Rape myth acceptance: exploration of its structure and its measurement using the Illinois rape myth acceptance scale. J Res Pers.

[74] Price EL, Byers ES, Belliveau N, Bonner R, Caron B, Doiron D, Greenough J, Guerette-Breau A, Hicks L, Landry A, Lavoie B, Layden-Oreto M, Legere L, Lemieux S, Lirette M, Maillet G, McMullin C, Moore R (1999). The attitudes towards dating violence scales: development and initial validation. J Fam Violence.

[75] Swartout KM, Flack Jr WF, Cook SL, Olson LN, Smith PH, White JW (2019). Measuring campus sexual misconduct and its context: the Administrator-Researcher Campus Climate Consortium (ARC3) survey. Psychol Trauma.

[76] Breitenbecher KH (2008). The convergent validities of two measures of dating behaviors related to risk for sexual victimization. J Interpers Violence.

[77] Macy RJ (2007). A coping theory framework toward preventing sexual revictimization. Aggress Violent Behav.

[78] Koss MP, Anderson R, Peterson ZD, Littleton H, Abbey A, Kowalski R, Thompson M, Canan S, White J, McCauley H, Orchowski L, Fedina L, Lopez E, Allen C (2024). The revised sexual experiences survey victimization version (SES-V): conceptualization, modifications, items and scoring. J Sex Res.

[79] Straus MA, Hamby SL, Boney-McCoy S, Sugarman DB (1996). The revised Conflict Tactics Scales (CTS2): development and preliminary psychometric data. Journal of Family Issues.

[80] Brown RL, Rounds LA (1995). Conjoint screening questionnaires for alcohol and other drug abuse: criterion validity in a primary care practice. Wis Med J.

[81] Kroenke K, Spitzer RL, Williams JB (2003). The Patient Health Questionnaire-2: validity of a two-item depression screener. Med Care.

[82] (2024). Certified peer support specialist and certified peer. Addiction Professionals of South Carolina.

[83] Recovery specialist manual. The Addictions Professionals of South Carolina (APSC).

[84] Flores EJ, Neil JM, Tiersma KM, Pappano CR, Mulligan C, Van Alphen MU, Park ER, Irwin KE (2021). Feasibility and acceptability of a collaborative lung cancer screening educational intervention tailored for individuals with serious mental illness. J Am Coll Radiol.

[85] Leon AC, Davis LL, Kraemer HC (2011). The role and interpretation of pilot studies in clinical research. J Psychiatr Res.

[86] NVivo version 14. Lumivero.

[87] Braun V, Clarke V (2006). Using thematic analysis in psychology. Qual Res Psychol.

[88] Diggle PJ, Heagerty P, Liang KY, Zeger SL (2002). Analysis of Longitudinal Data. 2nd edition.

[89] Molenberghs G, Verbeke G, Molenberghs G, Verbeke G (2005). The generalized linear mixed model (GLMM). Models for Discrete Longitudinal Data.

[90] Smith SG, Basile KC, Gilbert LK, Merrick MT, Patel N, Walling M, Jain A (2017). National intimate partner and sexual violence survey (NISVS): 2010-2012 state report. Centers for Disease Control and Prevention (CDC).

[91] SAMHSA's concept of trauma and guidance for a trauma-informed approach. Substance Abuse and Mental Health Services Administration.

